# Therapy-resistant septic olecranon bursitis due to *Mycobacterium gordonae*

**DOI:** 10.1051/sicotj/2016030

**Published:** 2016-11-29

**Authors:** Christian Konrads, Kilian Rückl, Mohammed El Tabbakh, Maximilian Rudert, Stefan Kircher, Piet Plumhoff

**Affiliations:** 1 Department of Orthopaedic Surgery, König-Ludwig-Haus, University of Würzburg Brettreichstr. 11 97074 Würzburg Germany; 2 Institute of Pathology, University of Würzburg Josef-Schneider-Str. 2 97080 Würzburg Germany

**Keywords:** Elbow, Bursa, Atypical bacteria, Infection, Antibiotics

## Abstract

*Introduction*: Septic olecranon bursitis due to atypical mycobacteria is rare. An insidious beginning can delay diagnosis and treatment. Antibacterial therapy recommendations are not well-defined for bursitis caused by atypical mycobacteria. We present a rare case of olecranon bursitis caused by *Mycobacterium gordonae*, reporting our experiences regarding pathogen identification and antibiotic therapy, which differs from regimes used in common septic bursitis mostly caused by *staphylococcus aureus*.

*Methods*: A 35-year-old male with bursitis olecrani received open bursectomy. Microbiological culture did not reveal bacteria. Due to wound healing complications revision surgery was performed four weeks postoperatively. Finally, *Mycobacterium gordonae* was identified by PCR and an antibiogram could be developed. A triple antimicrobial combination therapy with Rifampicin, Clarithromycin, and Ethambutol was administered systemically for 12 months. The patient was followed-up for 24 months.

*Results*: After the second operation with pathogen identification and antibiotic combination therapy the wound healed without any additional complications. At last follow-up 24 months after the first surgery with bursectomy and 23 months after revision surgery with debridement, the patient was still pain free with no significant clinical findings or tenderness to touch at the operation site. Elbow range of motion was full.

*Discussion*: As septic bursitis can be caused by many different and sometimes rare and difficult to identify bacteria, intraoperative probes should be taken and histopathological and microbiological analysis should be conducted, including PCR. In a young man with olecranon bursitis due to *Mycobacterium gordonae* surgical treatment and an antibiotic combination therapy showed a good clinical outcome after one and two years.

## Introduction

The olecranon bursa is a common site for bursitis. About two-thirds of the cases are aseptic and one-third septic. *Staphylococcus aureus* accounts for about 90% of septic olecranon bursitis; other pathogens found include Group-A-Streptococci, anaerobes, or parasites. Mycobacteria are very rarely found in septic bursitis [1].

Patients with septic olecranon bursitis usually have pain, fluctuant swelling, and sometimes fever. When atypical pathogens infect the olecranon bursa, the clinical picture usually is more subtle with an insidious beginning leading to delay in diagnosis and inappropriate treatment [2].

We report a case of olecranon bursitis caused by atypical mycobacteria in a patient with no other medical conditions.

## Case report

A 35-year-old healthy male was referred to our clinic with chronic olecranon bursitis of the right elbow after unsuccessful treatment with multiple corticosteroid injections.

The patient recalled no specific trauma. He had no systemic symptoms such as a fever for instance. On physical examination there was a fluctuant, slightly tender swelling with no redness or warmth over the olecranon process. Laboratory markers for infection (white blood cell count, erythrocyte sedimentation rate, and C-reactive protein) were all within normal range.

**Figure 1.**
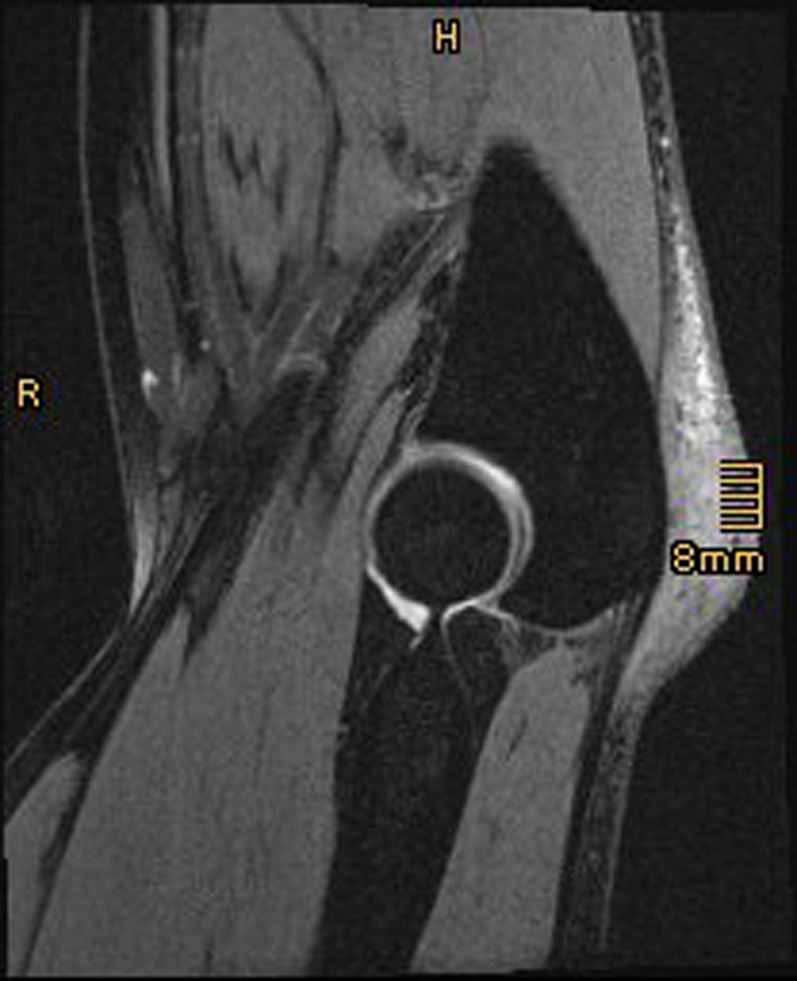
Sagittal T2-weighted MRI of a 35-year-old male patient’s right elbow showing olecranon bursitis with inhomogeneous signal enhancement of the thickened olecranon bursa.

Shortly before presentation in our outpatient clinic the patient had magnetic resonance imaging (MRI) of the elbow showing a thickened olecranon bursa with inhomogeneous signal enhancement in T2-weighted images (Figure 1). MRI did not show any bony or intraarticular pathologies. We conducted standard radiographs of the elbow joint (anteroposterior and lateral views). These did not show any pathologies. In particular, there was no bony olecranon spur apparent.

We decided to undertake a surgical debridement and we performed open olecranon bursectomy. Calculated intravenous antibiotic therapy with third-generation cephalosporin has been administered since tissue probes had been taken intraoperatively. Microbiological culture revealed no pathogen. The wound did not heal and had a persistent discharge and associated erythema in the margins of the incision. As the wound showed persistent secretion, we did a second surgical debridement four weeks after the first operation. Intraoperatively taken specimens were sent for histopathological and again bacteriological examinations, including polymerase chain reaction (PCR) were undertaken, looking for bacterial pathogens.

**Figure 2.**
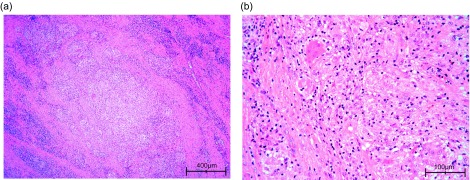
Microphotography of hematoxylin and eosin (HE)-stained bursa specimen. (a) Severe granulomatous inflammation (×100). (b) Higher magnification of non-caseating granulomas composed of epithelioid and multinucleated histiocytes (×400).

Histopathology (Figure 2) revealed acute and chronic inflammation. Bacterial identification in the tissue probes taken during the second surgery was successful in revealing rare acid-fast bacilli: *Mycobacterium gordonae*. Then the patient received three types of antibiotics according to the culture and sensitivity tests: namely Rifampicin, Clarithromycin, and Ethambutol. The wound healed without further complications. We successfully finished the triple antibiotic therapy after 12 continuous months. At this time the operation site did not show any problems. No further complications or revisions occurred. At last follow-up 24 months after the first operation and 23 months after the second surgery, the patient was still pain free with no significant clinical findings or tenderness to touch at the operation site. The patient had free elbow joint range of motion of 0/0/140°.

## Discussion

The presented patient had an infection of the olecranon bursa by *Mycobacterium gordonae*. This bacterium is very rarely found in septic bursitis [1]. After bursectomy a pathogen could not be identified at first. As the wound did not heal as expected, revision surgery was necessary and additional probes were taken intraoperatively. Eventually, pathogen identification was successful via PCR and microbiological cultivation finding *Mycobacterium gordonae*. This led to adequate antibiotic therapy and thus facilitated – in combination with surgical debridement – successful cure of the patient. Treating infections caused by atypical mycobacteria antibiotic therapy is recommended for at least 12 months continuously according to the German Reference Centre for Mycobacteria.

Atypical mycobacteria like *Mycobacterium gordonae* are present in the environment in soil and water [3]. Infections with atypical mycobacteria can occur due to trauma with skin abrasions leading to both skin and deep soft tissue infections [4]. Infection can also occur iatrogenically by injections into a bursa, which is primarily symptomatic due to chronic aseptic inflammation [2]. The presented patient did not recall any history of trauma, but had received several steroid injections into the affected olecranon bursa.

A review concluded that 38% of cases with nontuberculous mycobacterial olecranon bursitis had some sort of compromised immune system – for instance due to diabetes mellitus or intake of immunosuppressive drugs [2, 5, 6]. However, infections with atypical mycobacteria were also reported in young and healthy patients, in whom diagnosis and therapy were delayed due to absence of infection signs [2].

*Mycobacterium gordonae*, previously called *Mycobacterium aquae*, is a slow-growing mycobacterium that requires up to three weeks to reach mature growth. The best temperature for this microorganism to grow is between 35 °C and 37 °C. *Mycobacterium gordonae* could be cultivated from pipelines and freshwater [7].

Treatment for *Mycobacterium gordonae* induced septic bursitis and other infections caused by this atypical mycobacterium is not well established. Antimicrobial agents which have in vitro antibacterial effects against *Mycobacterium gordonae* include Rifampicin, Clarithromycin, Ethambutol, Fluoroquinolones, and Linezolid [7]. Most patients require surgical resection of the affected bursa for definitive treatment [4]. Endoscopic bursectomy might be a successful alternative to open surgery [8–10]. Probably, the penetration of the antimicrobial agents into the inflamed bursal tissue is suboptimal [2].

A case of olecranon bursitis with *Mycobacterium gordonae* infection was published, in which the antimicrobial therapy alone was sufficient for cure [3]. But it should be stated that this patient was diagnosed relatively early and did not receive corticosteroid injections.

The importance of antimicrobial therapy after resection is indefinite, although it is commonly used and it should be tailored according to the pathogen using proper in vitro susceptibility testing (antibiogram). Usually, the duration of antimicrobial therapy for infections caused by atypical mycobacteria is 12 months or longer [2]. It is recommended to use at least two antimicrobial agents simultaneously to decrease the probability of antibiotic resistance development [2].

## Conclusions

Septic olecranon bursitis due to the atypical *Mycobacterium gordonae* can be treated successfully by bursectomy and a triple antibiotic therapy over 12 months. For pathogen identification, we recommend histopathological and bacteriological examinations including polymerase chain reaction and long microbiological cultivation over four weeks.

## Conflict of interest

The authors declare that they have no conflict of interest.
